# Rare and Complicated Overlap of Stevens-Johnson Syndrome and Acute Generalized Exanthematous Pustulosis

**DOI:** 10.7759/cureus.15921

**Published:** 2021-06-25

**Authors:** Towfiqul A Chowdhury, Khandokar A Talib, Justin Patricia, Kennedy D Nye, Syed Ahmad Moosa

**Affiliations:** 1 Internal Medicine, United Health Services Wilson Medical Center, Johnson City, USA; 2 Internal Medicine, Upstate University Hospital, Syracuse, USA; 3 Surgery, Upstate University Hospital, Syracuse, USA; 4 Research, Bangladesh Medical Association of North America, New York, USA; 5 Internal Medicine, St. John's Episcopal Hospital, Queens Village, USA

**Keywords:** drug reaction, severe cutaneous adverse reaction, stevens-johnson syndrome, toxic epidermal necrolysis, acute generalized exanthematous pustulosis, overlap, skin biopsy

## Abstract

Stevens-Johnson syndrome (SJS)/toxic epidermal necrolysis (TEN) and acute generalized exanthematous pustulosis (AGEP) are two separate pathological entities of severe cutaneous adverse reactions (SCARs) with different etiologies and treatment strategies. Diagnosis is, however, complicated by the similarity in their clinical presentation. Although there are few claims of AGEP-SJS/TEN overlap, a simultaneous true overlap of SJS/TEN and AGEP has rarely been described in the literature.

Here, we report a case study of a 61-year-old female with a known allergy to sulfa drugs presenting with altered mental status, generalized weakness, and erythematous and excoriated purulent wounds. Based on initial workup and extensive consultation, the patient was diagnosed with severe sepsis secondary to diffuse purulent cellulitis, community-acquired pneumonia, and acute renal failure due to prerenal azotemia from dehydration. She was treated with several antibiotics, starting with vancomycin, piperacillin/tazobactam. Six days later, antibiotics were de-escalated to ceftriaxone and metronidazole because of the patient’s improved status. The medications were withheld when the patient started developing extensive blistering on day 8. Blood cultures ruled out any bacterial etiology. Skin biopsy confirmed overlapping features of AGEP and SJS/TEN. Due to the uncontrolled progression of her rash, she was transferred to the burn unit of a higher care center.

This is potentially the first histologically confirmed case of AGEP-SJS/TEN overlap in the United States. In this case study, a conclusive diagnosis would have never been made without a biopsy, especially because the condition presented clinically as SJS/TEN. We, therefore, recommend considering a potential overlap of multiple pathologies at each presentation or suspicion of a SCAR and performing an early skin biopsy in order to provide definitive diagnosis and treatment.

## Introduction

Adverse cutaneous drug reactions are a common occurrence in hospitalized patients, accounting for 2-3% of all inpatients. Few of these reactions develop into severe cutaneous adverse reactions (SCARs), affecting 0.1% of all hospital admissions [1]. Although some SCARs have different etiology, pathophysiology, and treatment, their clinical presentation can be similar. Two such SCARs are Stevens-Johnson syndrome (SJS)/toxic epidermal necrolysis (TEN) and acute generalized exanthematous pustulosis (AGEP). Both SJS/TEN and AGEP are mainly attributed to unfavorable drug reactions. As such, the two entities can be difficult to distinguish clinically and based on history. Therefore, histopathologic analysis is frequently needed for accurate diagnosis. However, a simultaneous overlap of SJS/TEN and AGEP is rare and was previously questioned to even exist at all. In addition to their similar presentation and potential overlap, their coexistence can create ambiguity, delay diagnosis, and skew clinical judgment [2,3].

In this article, we describe and discuss a rare case of an adult female with the clinical manifestations and histologically confirmed components of both SJS/TEN and AGEP.

## Case presentation

**Figure 1:**
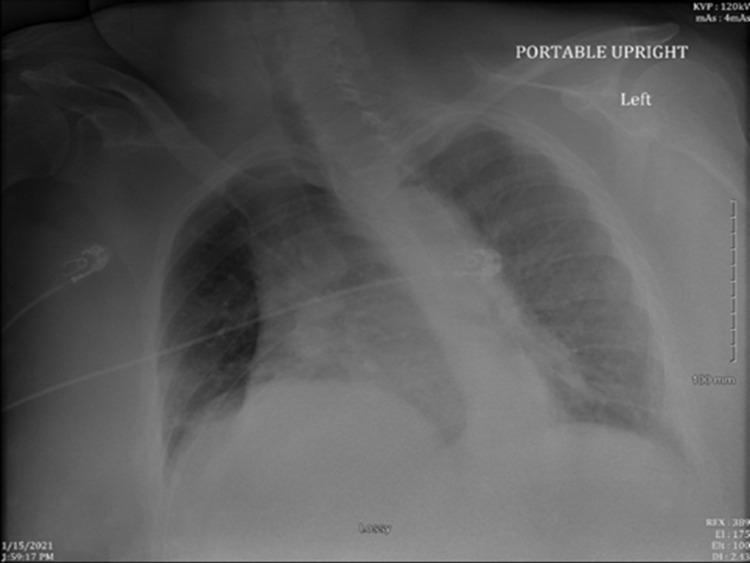
Chest X-ray with anteroposterior view revealing bilateral interstitial densities that suggest atelectasis or interstitial infiltrates.

**Figure 2:**
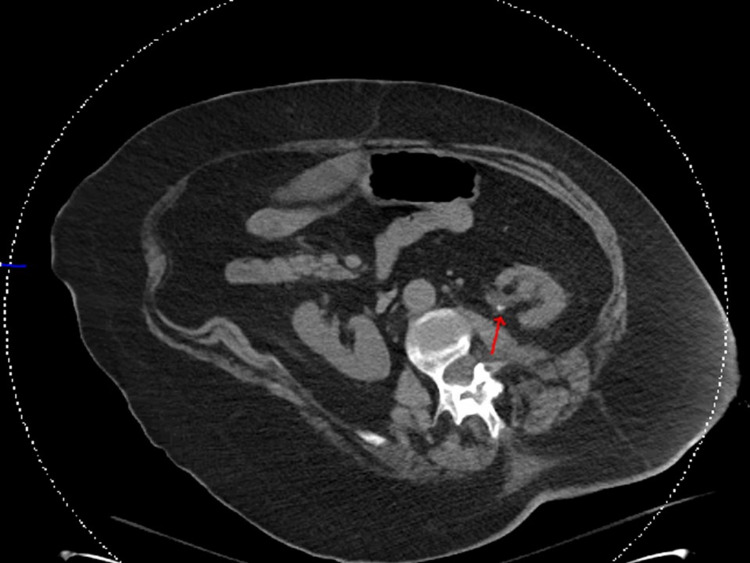
CT abdomen showing scattered punctate nonobstructive left renal calculi (red arrow).

**Figure 3:**
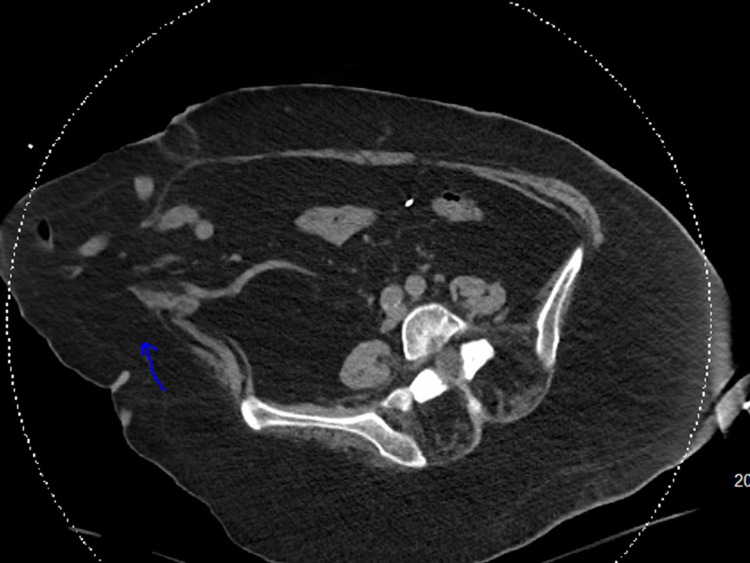
CT abdomen showing large right paramedian ventral hernia sac that contains abdominal contents (blue arrow).

A 61-year-old female with a past medical history of ulcerative colitis status post colectomy and colostomy presented to the emergency department (ED) with altered mental status and severe generalized weakness for an unknown duration. Upon arrival, the patient was alert but disoriented to person, place, and time, and unable to provide pertinent history. She was noted to be allergic to sulfa drugs and had not been following any primary care physician for the past 15 years with limited recent medical records available. Physical examination revealed erythematous and excoriated wounds draining purulent material over her left abdomen at the site of her colostomy bag and left superolateral thigh near the buttocks. Surgery was consulted for concern of possible necrotizing fasciitis at the site of her thigh wound, but it was determined that there was no surgical intervention needed with a recommendation for further resuscitative measures. Her vital signs on arrival revealed a blood pressure of 151/86 mmHg, pulse rate of 118 beats/minute, the temperature of 98.2 °F, respiratory rate of 29 breaths/minute, and oxygen saturation of 92% on 2 L nasal cannula. Initial laboratory results (Table 1) revealed the following: WBC 14.7 × 103/µL with a left shift, neutrophils 75%, blood urea nitrogen (BUN) 49 mg/dL, creatinine 2.1 mg/dL, glomerular filtration rate (GFR) 25 ml/min, HCO_3_ 13 mmol/l, AG 15, Cl 103 mmol/l, C-reactive protein (CRP) 18 g/dl, ABG: pH 7.278, pCO_2_ 26.5 mm Hg, pO_2_ 121 mm Hg, and bicarbonate 12.0 mmol/l on 3 L oxygen. Chest X-ray revealed bilateral airspace disease (Figure 1). Computed tomography (CT) of the abdomen (Figures 2 and 3) showed a fatty liver, sludge in the gallbladder, scattered punctate nonobstructive renal calculi, and a large right paramedian ventral hernia sac containing abdominal contents.

**Table 1:** Pertinent diagnostic workup of the patient during the hospital course.

	Admission	Day 6	Day 8	Day 13 (one day before transfer)	Reference values
Hemoglobin (g/dL)	15.5	13.6	13.5	11.1	12.2-15.5 g/dL
Leukocytes (10^3^/µL)	14.7	6	11.1	17	4.0-10.5 10^3^/µL
Neutrophils (10^3^/µL)	12.84	4	18.92	15	
Eosinophils (10^3^/µL)	0.03	0.3	0.33		<0.7 10^3^/µL
Lymphocytes (10^3^/µL)	0.80	0.8	0.99	0.5	1.4-4.0 10^3^/µL
Platelets (10^3^/µL)	408	240	197	177	125-425 10^3^/µL
Creatinine (mg/dL)	2.1	1.6	1.6	2.9	0.5-1.0 mg/dL
C-reactive protein (mg/dL)	18				<0.9 g/dL
Prothrombin time (seconds)	13.9				9-13 seconds
Activated partial thromboplastin time (seconds)	34				22-37 seconds

**Figure 4:**
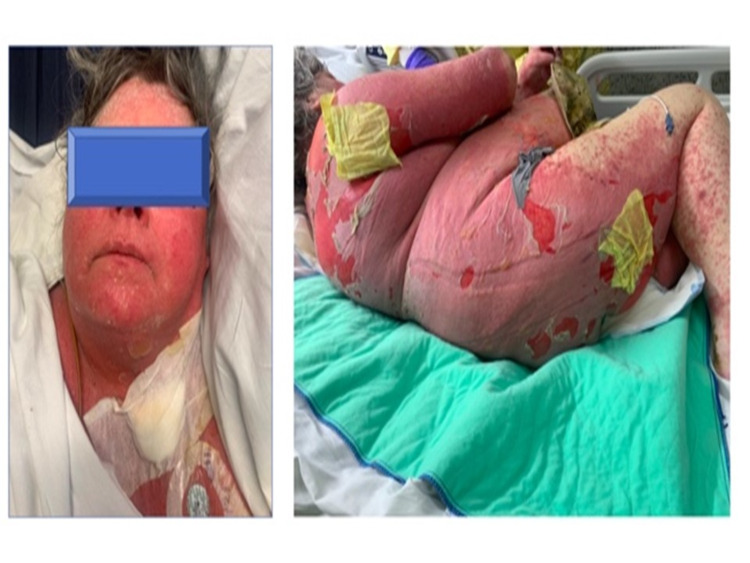
Fluid-filled blister on the face and rash with denuded skin involved more than 20% of the body surface.

**Figure 5:**
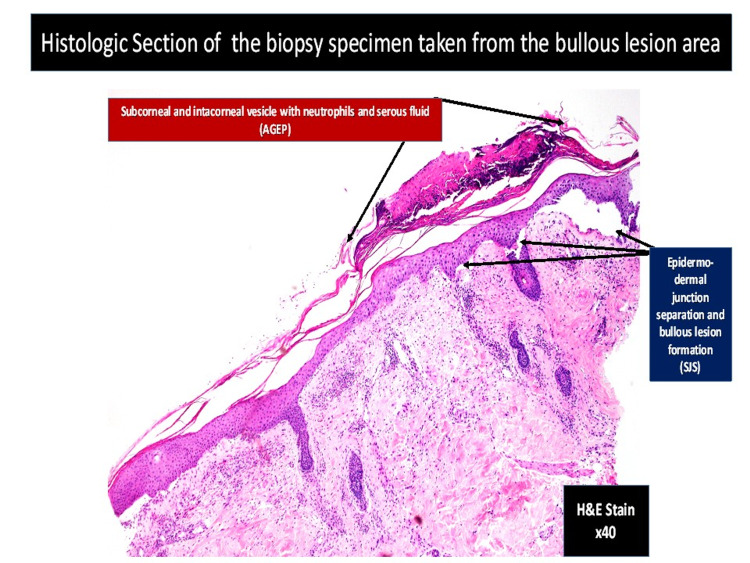
Light microscopy of bullous skin lesion (hematoxylin-eosin stain ×40) revealing subcorneal and intracorneal infiltration of neutrophil and serous fluid. There is epidermo-dermal junction separation with bullous formation.

**Figure 6:**
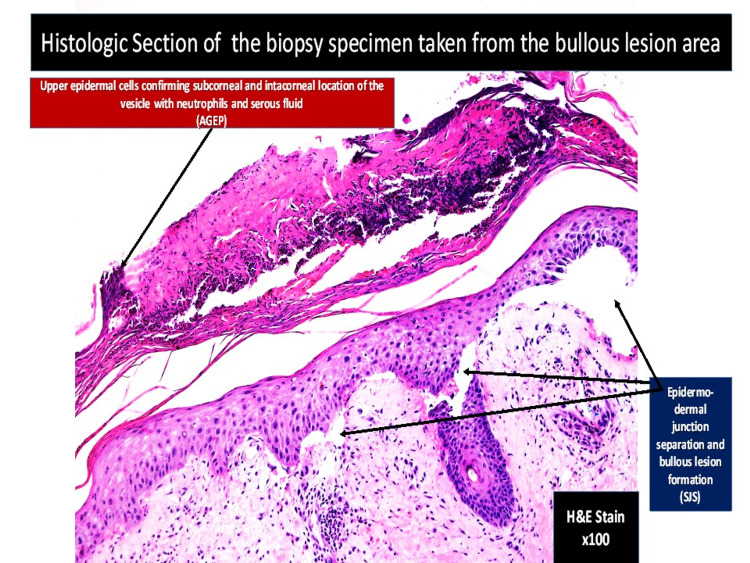
Light microscopy of bullous skin lesion (hematoxylin-eosin stain ×100) revealing upper epidermal cells confirming subcorneal confirming and intracorneal location of the vesicle with neutrophils and serous fluid (AGEP). AGEP: acute generalized exanthematous pustulosis.

**Figure 7:**
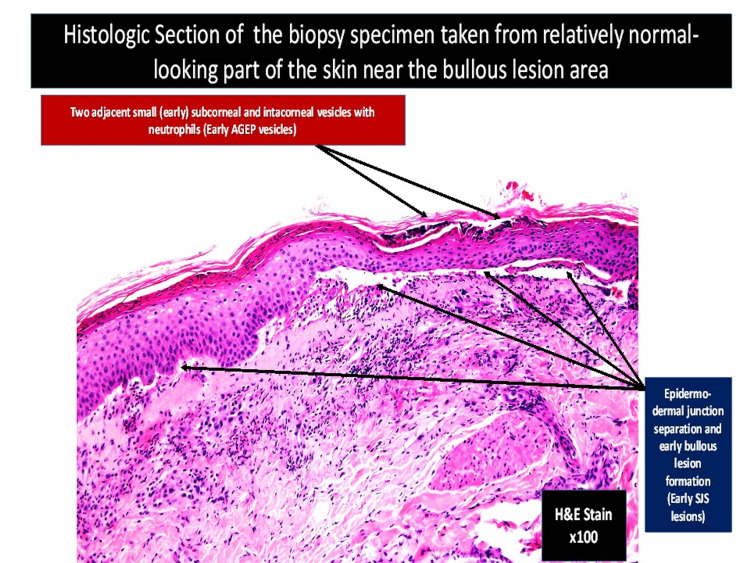
Light microscopy of bullous skin lesion (hematoxylin-eosin stain ×100) revealing two adjacent small (early) subcorneal and intracorneal vesicles with neutrophils (early AGEP vesicles). AGEP: acute generalized exanthematous pustulosis.

Blood cultures grew anaerobic Gram-positive cocci and *Staphylococcus aureus*. Culture from the abdomen wound revealed 2+ Gram-positive cocci and 1+ Gram-positive bacilli, while culture from the left thigh wound showed 3+ Gram-positive cocci, 3+ Gram-negative bacilli, and 1+ Gram-positive bacilli. She was diagnosed with severe sepsis secondary to diffuse extensive abdominal wall and bilateral lower extremity purulent cellulitis, community-acquired pneumonia, and acute renal failure due to prerenal azotemia from dehydration. Treatment was initiated in the ED with multiple intravenous antibiotics including piperacillin/tazobactam and vancomycin. She was subsequently admitted to a general medical floor and her antibiotics were changed to meropenem, doxycycline, and vancomycin. On day 6, owing to the improvement of her cellulitis and pneumonia, her antibiotics were de-escalated to ceftriaxone, metronidazole, and vancomycin per a recommendation from an infectious disease specialist. On day 8, she developed a maculopapular rash on the back of the thigh. Suspecting drug eruption, ceftriaxone was changed to ertapenem, and vancomycin and metronidazole remained unchanged. The rash continued to worsen, and blisters developed on the abdominal surface with denuded skin. At this time, there were multiple fluid-filled blisters on the abdominal wall and upper extremities. Some of the blisters ruptured and exposed the underlying tissue. Subsequently, all antibiotics were discontinued due to suspected SJS/TEN and intravenous methylprednisolone was started. As per the infectious disease specialist, daptomycin and metronidazole were restarted for prophylactic coverage, although repeat blood cultures did not reveal any growth. The rash continued to worsen, involving more than 20% of body surface area (BSA) with denuded skin. Blisters spread to the face, abdominal surface, and upper extremities, but spared the oral mucosa (Figure 4). Meanwhile, the patient developed oliguric renal failure due to suspected acute interstitial nephritis and required hemodialysis. Skin biopsy revealed overlapping features of AGEP and SJS/TEN (Figures 5-7). Due to the uncontrolled progression of her rash, she was transferred to the burn unit of a higher care center.

## Discussion

SJS and TEN represent opposite ends of a single SCAR that presents along a spectrum of severity. This condition is characterized by a rare blistering drug reaction that causes detachment of the entire epidermis. In 80% of cases, it is caused by drugs, particularly antibiotics, antiepileptics, allopurinol, sulfasalazine, and certain NSAIDs [4,5]. The pathophysiology of SJS/TEN, although not understood completely, is thought to be an immune-mediated reaction to medications that result in damage to keratinocytes, blistering, and necrosis of the entire epidermis [6]. The percentage of BSA involvement points to a diagnosis in this spectrum: <10% BSA affected in SJS, 10-30% BSA affected in SJS/TEN overlap, and >30% BSA affected in TEN. Clinically, cutaneous manifestations of SJS/TEN may be preceded by fever, malaise, pharyngitis, arthralgias, and fatigue [7]. These are followed by painful, erythematous macules, Nikolsky-positive blisters, epidermal necrosis, detachment, and sloughing one to three weeks after the offending drug is administered. One key feature that distinguishes SJS/TEN from certain other SCARs is that mucous membranes are very frequently involved. SJS/TEN is mainly diagnosed based on presentation and medication history; however, biopsy and histopathological analysis confirm the diagnosis by showing keratinocyte necrosis and involvement of the entire epidermal layer. A biopsy is, therefore, an important tool to help distinguish SJS/TEN from other disease entities [2]. Treatment involves discontinuing the offending drug and supportive measures such as wound management, fluid replacement, antibiotics if there is superimposed infection, and addressing any potential complications.

AGEP is another SCAR that can present similarly and potentially even overlap with SJS/TEN despite being distinct clinical entities. The onset of AGEP is often within one to three days after administration of the offending drug. AGEP often presents with fever and an eruption of numerous, small, sterile non-follicular pustules with extensive background erythema that frequently involves the face and intertriginous areas. Mucosal involvement is less frequent in AGEP than in SJS/TEN. The coalescence of multiple areas of these pustules can sometimes present with cutaneous sloughing and epidermal erosion, creating a similar presentation to SJS/TEN despite the etiology being different. These cutaneous manifestations are often accompanied by neutrophilia and systemic symptoms [2]. AGEP is commonly elicited by antibiotics and the calcium channel blocker diltiazem [8]. The exact mechanism and pathophysiology behind AGEP remain unclear; however, it is thought to be due to significant T-cell and neutrophil recruitment and activation. AGEP frequently displays a milder clinical course than SJS/TEN and can resolve sooner after discontinuation of the offending drug, often within 15 days. Diagnosis can be established by the use of the EuroSCAR validation score. To confirm the diagnosis, a biopsy should be performed. Histopathology examination shows intraepidermal or subcorneal pustules with lymphocytic dermal infiltrate and edema [2]. Management involves removing the offending drug and implementing supportive measures such as fluids and pain management. With proper management, AGEP often resolves without any major sequelae.

AGEP and SJS/TEN are both in the spectrum of SCARs that also includes Drug Rash with Eosinophilia and Systemic Symptoms (DRESS) syndrome. These conditions are most commonly attributed to unfavorable drug reactions. Although the etiology of this spectrum is not fully understood, genetic, immunological, and environmental factors have been implicated. These diseases can be classified as type IV hypersensitivity reactions. In the sensitization phase, an antigen is introduced into the skin, taken up by Langerhans cells, and migrated to the lymph nodes where T lymphocytes are sensitized to the antigen. Repeated contact with the antigen triggers the sensitized T lymphocytes to secrete lymphokines and cytokines, such as IFN-gamma, and TNF-alpha, which elicit an inflammatory reaction within the tissue.

Although cytokines and their definitive pathophysiological roles in both AGEP and SJS/TEN are still being researched, the literature classifies type IV hypersensitivity reactions based on their cytokine involvement [9,10]. Based on this classification, SCARs can be further broken down into type IV hypersensitivity subcategories. SJS/TEN, for example, is mediated via cytotoxic T lymphocytes, making it a type IVc hypersensitivity reaction [9,10]. These cells are activated by drug antigens and secrete perforin and granzyme cytokines that exert direct toxic effects. These toxic effects result in forming maculopapular and bullous exanthema, the type of skin reaction clinically observed in SJS/TEN. AGEP is also mediated by T lymphocytes; however, these cells exert their effect through IL-8 cytokine secretion, making it a type IVd hypersensitivity reaction [9,10]. This recruits neutrophils to the reaction site, leading to pustular exanthema reactions that are commonly associated with AGEP. Table 2 compares and contrasts the two pathologies.

**Table 2:** Features of SJS/TEN versus AGEP versus our patient. SJS/TEN: Stevens-Johnson syndrome/toxic epidermal necrolysis, AGEP: acute generalized exanthematous pustulosis.

	SJS/TEN	AGEP	Patient
Histology	Full-thickness epidermal necrosis with perivascular eosinophilic infiltration.	Intraepidermal/sub-corneal pustules with lymphocytic dermal infiltrate and edema.	Sub/intracorneal vesicles with neutrophils and serous fluid, epidermal-dermal separation, and bullous lesion formation.
Classification of hypersensitivity reaction	IV	IV	IV
Clinical Manifestations	Fever and constitutional symptoms with painful erythematous macules and Nikolsky-Positive blisters one to three weeks after offending medication, involving mucous membranes.	Fever and eruption of numerous, small sterile non-follicular pustules on an erythematous background, frequently in the intertriginous areas often without mucous membrane involvement.	Erythematous macules and Nikolsky-positive blisters with numerous small pustules involving the intertriginous areas, with sloughing and mucous membrane involvement as well.

The true overlap of SCARs rarely occurs and was previously questioned to even exist at all. The symptomatic overlap creates ambiguity, which can delay diagnosis and skew clinical judgment [2,3]. Bouvresse et al. retrospectively analyzed 216 cases with SCARs using regiSCAR algorithms and found that true overlap represented about 2% of the analyzed cases. In their study, 45 of the 216 SCARs were deemed to have some features of clinical overlap, but when verified through the regiSCAR algorithm, only 3 of the 45 were confirmed overlap: two cases of SJS-TEN/DRESS, and one case of AGEP/DRESS, but no AGEP-SJS/TEN overlap [11]. An online English literature search since this study has not turned up any cases with definitive histological evidence of true AGEP-SJS/TEN overlap. There are no agreed-upon diagnostic criteria for SJS/TEN. Although common clinical features are frequently used for diagnosis, it is never confirmed without histological evidence. AGEP, on the other hand, requires examination of a skin biopsy for diagnosis. So, with this information, claimed cases of overlap do not usually qualify as true overlap diagnosis, and actual histological evidence of SCAR overlap is rare.

To the best of our knowledge, we present a case that could be the first histologically confirmed case of AGEP-SJS/TEN overlap in the United States. Although the patient clinically presented with several common features of SJS/TEN, such as hemodynamic instability and sloughing lesions with mucosal involvement, skin biopsy demonstrated definitive histological features of both AGEP and SJS/TEN. Since severe cases of AGEP can mimic SJS/TEN, clinicians should be cautious to use clinical evaluation alone when evaluating patients for SCARs. Immediate biopsy to determine a conclusive diagnosis in SCARs can prevent delay in treatment.

The first suspicion of a SCAR should warrant a skin biopsy to definitively determine the etiology for this SCAR. Establishing the etiology is an important step for a correct diagnosis since clinical symptoms alone are not reliable for SCAR diagnosis [12-14]. Treatment of SCARs begins with discontinuation of the suspected offending drug, but further management should be tailored to the specific reaction with prompt diagnosis. Minimal data on decisive additional treatment recommendations for SCARs are available. Corticosteroids and intravenous immunoglobulins have been used with some efficacy and experimental therapy, such as cyclosporine, is still under investigation [15]. Without performing a skin biopsy, treatment is not based upon a definitive diagnosis.

Biopsy should always be done as part of the standard workup of any SCAR. For example, in this case, a conclusive diagnosis would have never been made without a biopsy, especially because it clinically presented as SJS/TEN. Further studies on the treatment of proven histologically diagnosed SCARs could help better tailor future management of these conditions.

## Conclusions

SCARs are a group of fatal conditions that are usually related to drug reactions. They involve the skin and mucous membranes. Overlap of these conditions is a rare occurrence. However, it should be considered at each presentation or suspicion of a SCAR and confirmed with a skin biopsy in order to provide definitive diagnosis and treatment. 

## References

[1] Nayak S, Acharjya B (2008). Adverse cutaneous drug reaction. Indian J Dermatol.

[2] van Hattem S, Beerthuizen GI, Kardaun SH (2014). Severe flucloxacillin-induced acute generalized exanthematous pustulosis (AGEP), with toxic epidermal necrolysis (TEN)-like features: does overlap between AGEP and TEN exist? Clinical report and review of the literature. Br J Dermatol.

[3] Meiss F, Helmbold P, Meykadeh N, Gaber G, Marsch WC, Fischer M (2007). Overlap of acute generalized exanthematous pustulosis and toxic epidermal necrolysis: response to antitumour necrosis factor-alpha antibody infliximab: report of three cases. J Eur Acad Dermatol Venereol.

[4] Sassolas B, Haddad C, Mockenhaupt M (2010). ALDEN, an algorithm for assessment of drug causality in Stevens-Johnson Syndrome and toxic epidermal necrolysis: comparison with case-control analysis. Clin Pharmacol Ther.

[5] Mockenhaupt M, Viboud C, Dunant A (2008). Stevens-Johnson syndrome and toxic epidermal necrolysis: assessment of medication risks with emphasis on recently marketed drugs. The EuroSCAR-study. J Invest Dermatol.

[6] Stern RS, Divito SJ (2017). Stevens-Johnson syndrome and toxic epidermal necrolysis: associations, outcomes, and pathobiology-thirty years of progress but still much to be done. J Invest Dermatol.

[7] Roujeau JC, Stern RS (1994). Severe adverse cutaneous reactions to drugs. N Engl J Med.

[8] Sidoroff A, Dunant A, Viboud C (2007). Risk factors for acute generalized exanthematous pustulosis (AGEP)-results of a multinational case-control study (EuroSCAR). Br J Dermatol.

[9] Szatkowski J, Schwartz RA (2015). Acute generalized exanthematous pustulosis (AGEP): a review and update. J Am Acad Dermatol.

[10] Pichler WJ (2003). Delayed drug hypersensitivity reactions. Ann Intern Med.

[11] Bouvresse S, Valeyrie-Allanore L, Ortonne N (2012). Toxic epidermal necrolysis, DRESS, AGEP: do overlap cases exist?. Orphanet J Rare Dis.

[12] Tajmir-Riahi A, Wörl P, Harrer T, Schliep S, Schuler G, Simon M (2017). Life-threatening atypical case of acute generalized exanthematous pustulosis. Int Arch Allergy Immunol.

[13] McDonald KA, Pierscianowski TA (2017). A case of amoxicillin-induced acute generalized exanthematous pustulosis presenting as septic shock. J Cutan Med Surg.

[14] Mawri S, Jain T, Shah J, Hurst G, Swiderek J (2015). Vancomycin-induced acute generalized exanthematous pustulosis (AGEP) masquerading septic shock-an unusual presentation of a rare disease. J Intensive Care.

[15] Lerch M, Mainetti C, Terziroli Beretta-Piccoli B, Harr T (2018). Current perspectives on Stevens-Johnson syndrome and toxic epidermal necrolysis. Clin Rev Allergy Immunol.

